# The Fatal Circle of NETs and NET-Associated DAMPs Contributing to Organ Dysfunction

**DOI:** 10.3390/cells11121919

**Published:** 2022-06-14

**Authors:** Helena Block, Jan Rossaint, Alexander Zarbock

**Affiliations:** Department of Anesthesiology, Intensive Care and Pain Medicine, University Hospital Muenster, 48149 Muenster, Germany; helena.block@uni-muenster.de (H.B.); rossaint@uni-muenster.de (J.R.)

**Keywords:** neutrophil extracellular traps, damage associated molecular pattern, inflammation, innate immune response, remote organ damage, HMGB1, LL37, histone, cfDNA, CIRP

## Abstract

The innate immune system is the first line of defense against invading pathogens or sterile injuries. Pattern recognition receptors (PRR) sense molecules released from inflamed or damaged cells, or foreign molecules resulting from invading pathogens. PRRs can in turn induce inflammatory responses, comprising the generation of cytokines or chemokines, which further induce immune cell recruitment. Neutrophils represent an essential factor in the early immune response and fulfill numerous tasks to fight infection or heal injuries. The release of neutrophil extracellular traps (NETs) is part of it and was originally attributed to the capture and elimination of pathogens. In the last decade studies revealed a detrimental role of NETs during several diseases, often correlated with an exaggerated immune response. Overwhelming inflammation in single organs can induce remote organ damage, thereby further perpetuating release of inflammatory molecules. Here, we review recent findings regarding damage-associated molecular patterns (DAMPs) which are able to induce NET formation, as well as NET components known to act as DAMPs, generating a putative fatal circle of inflammation contributing to organ damage and sequentially occurring remote organ injury.

## 1. Introduction

The essential task of the immune system is the recognition of potential danger, invaders, or injuries to the organism, and to respond adequately to eliminate, fight or repair the respective threats. The innate immune response acts as a first-line defense, and is largely dependent on immune cells switching the organism into an inflammatory state. Sensing of threats occurs through pattern-recognition receptors (PRRs), which recognize both pathogen-associated molecular patterns (PAMPs), which are exogenous, microorganism-derived molecules, as well as damage-associated molecular patterns (DAMPs), consisting of endogenous and exogenous non-microbial molecules [1]. PRRs are a heterogeneous family with four major subfamilies, comprising Toll-like receptors (TLRs), cytoplasmic nucleotide-binding and oligomerization domain (NOD)-like receptors (NLRs), retinoic acid-inducible gene 1 (RIG1)-like receptors, and C-type lectin receptors (CLRs) [2,3]. However, non-PRRs are also able to recognize PAMPs, such as the receptor for advanced glycation end products (RAGE), integrins, CD91, and CD44 [4]. They initialize a tightly regulated immune response consisting of the production of pro-inflammatory cytokines such as tumor necrosis factor alpha (TNFα), or interleukin 1 (IL1), reactive oxygen species (ROS), nitric oxide (NO), vasoactive amines (e.g., histamine, serotonine), neuropeptides, and arachidonic acid metabolites (prostaglandins, leukotrienes). Furthermore, the inflammatory response includes the activation of platelets and also increases vascular permeability [5]. Platelets are important players in the early immune response, expressing PRRs such as TLR2 and TLR4 [6]. They are capable of secreting chemokines, cytokines, and other inflammatory mediators [7], and further contribute to leukocyte recruitment, activation, and emigration into inflamed tissues [8]. In addition, monocytes and macrophages essentially contribute to the production of cytokines, lipid mediators, reactive oxygen or nitrogen species, and release anti-microbial enzymes and peptides. Furthermore, monocytes and macrophages are also involved in anti-inflammatory processes to prevent uncontrolled activation of the immune system [9]. This tight regulation is a prerequisite for an appropriate immune response. A maladaptive response causes systemic inflammation, organ injury, and a disturbed resolution process, and may end in death. This review will summarize the role of neutrophil extracellular traps (NETs), their components acting as DAMPs, and DAMPs triggering NET formation during inflammation. Additionally, it provides an overview of the influence of NET formation on kidney, lung, or liver injuries, and its contribution to immunothrombotic events.

## 2. Mechanisms of Extracellular Trap Formation and Clearance

The release of pro-inflammatory mediators during the innate immune response results in leukocyte recruitment to the inflammatory site. The intimate contact of immune cells, such as neutrophils, with the inflamed endothelium results in the activation of different signaling cascades [10]. Activated neutrophils fulfil numerous tasks to fight infection, such as the production of ROS, phagocytosis, degranulation, and the release of neutrophil extracellular traps (NETs). NETs are comprised of decondensed chromatin decorated with a variety of proteins, e.g., neutrophil elastase (NE), myeloperoxidase (MPO), histones, cathelicidins, α-defensins, calprotectin, and cytoskeletal proteins [11,12], whereas a stimulus-dependent protein composition is likely [13,14]. To date, many stimuli have been shown to induce NET formation. NET release can be stimulated via TLRs, G protein-coupled receptors (GPCRs), chemokine and cytokine receptors, Fc receptors (FcRs), and β_2_-integrins [15]. Two main pathways in neutrophils have been described: lytic NET formation, and non-lytic NET formation, but other forms of extracellular traps also exist. 

### 2.1. Lytic or Suicidal NET Formation

**Figure 1:**
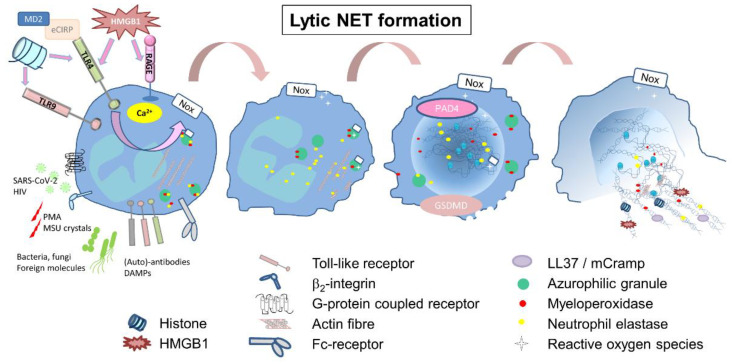
Inducers and mechanism of the lytic NET formation. Gram-negative bacteria and bacteria-deriving molecules, antibodies, phorbol-12-myristat-13-acetat (PMA), monosodium urate crystals (MSU) or damage-associated molecular patterns (DAMPs) are capable of activating neutrophils via different receptors and initiating NET formation. Histones induce NET formation via the toll-like receptors (TLR) 4 and 9, eCIRP via parallel binding of myeloid differentiation factor 2 (MD2) and TLR4, and high-mobility group box 1 (HMGB1) via RAGE and TLR4. Calcium is released into the cytosol, followed by activation of the NADPH-oxidase complex (NOX), which generates reactive oxygen species (ROS). In a ROS-dependent step, neutrophil elastase (NE) gets released from the membranes of azurophilic granules and translocates into the nucleus and in parallel degrading actin fibers. NE activity induces the decondensation of chromatin, further supported by the PAD4-dependent citrullination of histones. The activation of gasdermin D (GSDMD) leads to the formation of pores in the cell membrane, thereby enabling the release of chromatin, which has been decorated with cytosolic or granule-associated molecules such as histones, LL37, HMGB1, MPO and NE into the environment.

To date, most of the currently confirmed inducers of NET formation, such as Gram-negative bacteria, fungi, viruses, PMA, monosodium urate crystals (MSU), or bacterial molecules, initiate the lytic pathway, resulting in the death of the cell. In response to those stimuli, infectious, as well as sterile, calcium is released from the endoplasmatic reticulum into the cytosol, resulting in NADPH-dependent ROS production, implicating protein kinase C (PKC) or the RAF-MEK-MAPK pathways [16,17]. This signaling further initiates the dissociation of neutrophil elastase (NE) from a membrane-associated complex into the cytosol and activates its proteolytic activity in a myeloperoxidase—(MPO) dependent manner (Figure 1). To arrest actin dynamics, NE degrades F-actin and translocates into the nucleus, where NE and MPO drive chromatin decondensation and histone cleavage [18,19], further supported by peptidylarginine deiminase 4 (PAD4)-dependent histone citrullination [20]. However, NADPH-, NE- and PAD4-independent pathways have been described, too [21,22,23]. Cell cycle proteins [24] support nuclear envelope breakdown followed by the release of chromatin into the cytosol, where nuclear and cytosolic proteins are mixed [25]. The liberation of NET fibers involves gasdermin D (GSDMD), forming pores in granule and plasma membranes [26,27]. This kind of NET formation occurs in a time frame of up to eight hours, ends up with cell death and is often described as lytic NET release or suicidal NETosis (Figure 1). 

### 2.2. Non-Lytic or Vital NET Formation

**Figure 2:**
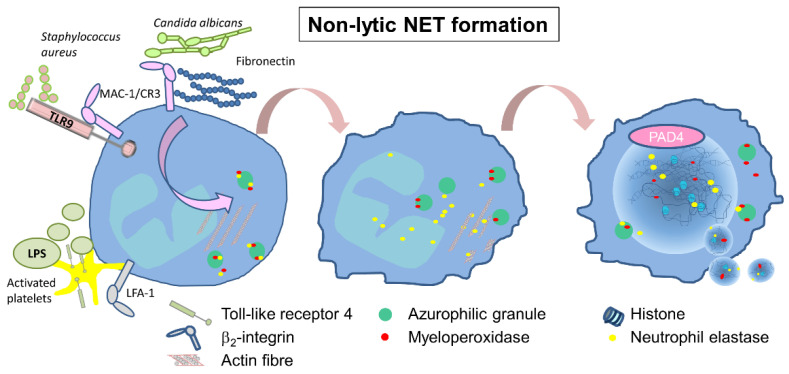
Inducers and mechanism of non-lytic NET formation. To date, only *S. aureus*, *C. albicans*, and activated platelets have been proven to induce early non-lytic NET formation in neutrophils. *C. albicans* induces NET formation via MAC1-signaling in presence of fibronectin, *S. aureus* via TLR9 and MAC1, and activated platelets require LFA-1 and LPS. During early NET formation, NE and MPO are released in an NADPH-independent manner into the cytosol and the nucleus, resulting the decondensation of chromatin, supported by PAD4-activity. Nuclear DNA fibers are finally released into the cytosol via vesicles, leaving an anucleated but functional cytoplast.

Early non-lytic NET release has been described for only a small number of stimuli. It was observed for neutrophils in close contact with activated platelets [28,29] or in response to *Staphylococcus aureus* [30] and *Candida albicans* infections [31,32] (Figure 2). The pathogen-induced response has been shown to depend on TLRs and/or the complement receptors [30,31], whereas platelet-induced NET formation during infection occurs in an LFA1-dependent manner, and depends on the direct interaction of neutrophils and platelets [29]. It takes place rapidly after 5–60 min of stimulation, and is independent of the NADPH oxidase pathway [33]. It also involves the translocation of NE to the nucleus, histone citrullination, and chromatin decondensation [34], as demonstrated following stimulation with *C. albicans* [18,19], but the membrane does not disintegrate, and the protein-decorated chromatin is released via vesicles [30] (Figure 2). Even the remnants of non-lytic NET formation, cytoplasts, are able to keep their mobility and fulfill important functions, such as phagocytosis, the activation of dendritic cells, and the release of cytotoxic molecules [30,35,36].

### 2.3. Other Forms of Extracellular Trap Formation

Besides the release of extracellular traps of nuclear origin, eosinophils as well as neutrophils are able to release mitochondrial DNA (mtDNA). Neutrophils primed with granulocyte-macrophage colony-stimulating factor followed by stimulation with a TLR4 agonist or C5a have been shown to release mtDNA [37]. Similarly, eosinophils primed with IL5 or IFNγ and stimulated with LPS expelled mitochondrial DNA [38]. In contrast to NETs, the mtDNA traps are not decorated with histones or antimicrobial granule proteins, thus complicating their identification, and further questioning their role as potential pathogen defense mechanism. Additionally, the release of nuclear DNA by macrophages or monocytes has been described by different groups and is termed as macrophage extracellular traps (METs). They are also considered to offer anti-microbial functions and contribute to pathology, as has been reviewed in detail elsewhere [39]. 

### 2.4. Degradation or Anti-Inflammatory Properties of NETs

Little is known about the removal of NETs. Besides the degradation of NETs through DNases, some studies also suggest a contribution of macrophages to NET elimination by resolution and degradation [40]. In vitro experiments with human monocyte-derived macrophages and PMA-stimulated human neutrophils demonstrated that macrophages are able to internalize NETs in a cathelicidin LL37-dependent manner and degrade DNA via TREX1/DNAseIII. In this setting, dendritic cells contribute to extracellular NET degradation by providing DNase1L3 [41]. In contrast, Apel and colleagues revealed a mechanism where phagocytosed NETs activate the innate immune sensor cyclic GMP-AMP synthase, thereby inducing the production of pro-inflammatory type I interferons [42]. Another study suggested a two-phase model of macrophages: in the early phase, M2 macrophages induce a pro-inflammatory response and sustain the inflammatory state, whereas in the second phase, M1 macrophages undergo cell death with nuclear decondensation in a PAD4-dependent manner, resulting in the local release of extracellular DNA. In the late phase, M1 macrophages degrade DNA in a caspase-activated DNase-dependent manner, resulting in the clearance of extracellular DNA within 24 h [43]. 

Studies describing anti-inflammatory properties of NETs are scarce. To date, only NET aggregates (aggNETs), which are formed at sites of high neutrophil density, have been suggested to act in an anti-inflammatory capacity, since they have been shown to sequester and degrade histones further attenuating their cytotoxic effect on epithelial cells [44]. This process was executed by at least two aggNET-borne serine proteases, NE and proteinase 3 (PR3). Furthermore, they are capable of resolving inflammation by the proteolytical degradation of inflammatory cytokines and chemokines [45,46]. Nevertheless, the physiological relevance of these proposed mechanisms remains elusive, and further work is required to shed light on the mechanisms of NET resolution and degradation.

## 3. DAMPs Associated with NETs or Capable of Inducing NETs

**Figure 3:**
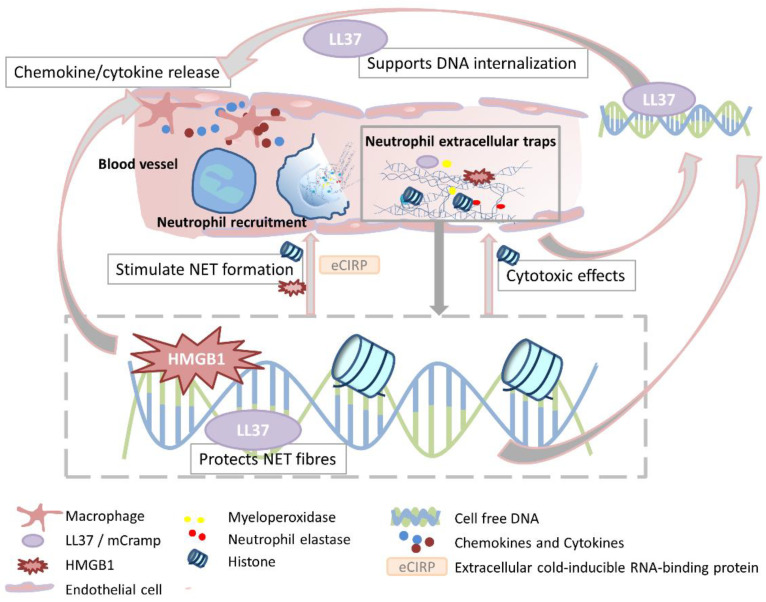
Schematic illustration of NET-associated DAMPs contributing to inflammation. During inflammation, resident cells such as macrophages or endothelial cells lining the vessel release pro-inflammatory cytokines and chemokines, inducing neutrophil recruitment. Activated neutrophils may release NETs decorated with diverse proteins such as neutrophil elastase, myeloperoxidase, LL37 or mCramp in murine cells, HMGB1, and histones. Some of them have been reported to act as DAMPs. Arrows indicate the contribution of the respective proteins to the pro-inflammatory features. HMGB1 induces NET formation, as well as cytokine/chemokine release. LL37 protects NET fibers from degradation, and also facilitates the internalization of cfDNA, which in turn also promotes cytokine release. Histones have cytotoxic effects on endothelial cells, resulting in additional cfDNA, and are also capable of directly inducing NET formation. The molecule eCIRP has recently been described as promoting NET formation.

During inflammation, danger signals initiate the immune response, resulting in the recruitment of immune cells to fulfill the appropriate function for antagonizing the triggering insults. Several studies have identified DAMPs that can induce NET formation. Interestingly, some proteins decorated on NETs may function as DAMPs, resulting in enhanced cytokine production and therefore enhanced neutrophil recruitment and activation. This may result in a fatal circle (Figure 3) of persisting inflammation, which may further end in organ damage, systemic inflammation, organ failure, or death. A brief summary of those molecules addressing these criteria are listed in the following Table 1 and are consecutively described in more detail.

### 3.1. Histones

Histones are usually located in the nucleus, complexed with DNA to form the nucleosome [71]. They can be released either passively during cell death, or actively during NET formation or vesicle release, as observed for LPS-challenged murine macrophages [72,73]. Their direct cytotoxicity has been demonstrated in vitro for endothelial cells and in vivo in murine models of lipopolysaccharide- (LPS-) or cecal ligation and puncture (CLP)-induced sepsis [74]. Other studies revealed that NET-induced cytotoxic effects on human alveolar epithelial cells were reduced upon treatment with anti-histone antibodies [75]. Furthermore, it has been shown that sublethal application of histones to mice induces high levels of cytokines such as TNFα, IL6, and IL10 in a TLR4- but not TLR2-dependent manner. In contrast, in vitro experiments revealed that histones are able to signal via both TLR2 and TLR4 [47]. In line with this, histone levels in septic patients are significantly increased. Applying sera of these patients to cardiomyocytes ex vivo induced cell death, which was abrogated by antibody-histone depletion [76]. Interestingly, studies suggest a role for histones as NET inducers. During acute kidney injury, histones released from necrotic cells induced NET formation, further accelerating kidney damage, promoting inflammation, and triggering remote organ injury in the lungs through TLRs [48]. Following ischemia–reperfusion injury (IRI) in the murine liver, a dose-dependent increase in NET-specific markers in response to histones was observed. This effect was dependent on TLR4 and TLR9 on neutrophils. These authors demonstrated that histones released from stressed hepatocytes stimulate neutrophils to form NETs to exacerbate liver damage [49]. In both cases, anti-histone treatment was effective in reducing injury severity. Taken together, these data indicate that histones are part of a pro-inflammatory positive feedback loop of damage. Their passive release through cell death initiates the PRR-induced immune cell recruitment, induces NET formation, subsequently leading to the active release of more histones, whose cytotoxic activity further potentiate local damage. A possible inflammation-limiting intervention in this loop is the presence of fibrinogen, which is able to reduce the cytotoxicity through binding to histones, and additionally delay further NET formation in a β_2_-integrin-dependent manner [77]. Fibrinogen depletion or consumption, when it occurs during sepsis or trauma, might accordingly contribute to the maladaptive overwhelming immune response.

### 3.2. Cell Free DNA (cfDNA)

Cell free nuclear DNA in the extracellular space can either be foreign, originating from invaders such as bacteria and viruses, or derive from the host itself through apoptosis, necroptosis, pyroptosis, or NET formation. Additionally, mitochondrial DNA can be released, as well, for instance during sepsis or trauma. Both nuclear and mitochondrial DNA have been proven to act as DAMPs [78,79]. Despite the origin of these different cfDNAs, they can all function as DAMPs and initiate multiple pro-inflammatory cascades (Figure 3). Elevated levels of cfDNA have been found in septic patients, as well as in patients with various autoimmune diseases [80]. Besides promoting the release of pro-inflammatory cytokines, it contributes to sustained inflammation by prolonging the life span of neutrophils [79]. Interestingly, there is a spatial segregation between cfDNA and its respective signaling receptors TLR9, cGAS, IFI16, AIM2, or STING, which are located intracellularly and are able to initiate immune responses [50]. This separation is likely related to their usual function in recognizing nucleic acids resulting from infectious insults by bacteria or viruses. An intracellular location of receptors may also prevent inadvertent stimulation by extracellular host DNA, since the uptake of DNA into the cytosol has to be supported actively. One possible mechanism of active DNA delivery to the cytosol in the context of inflammation is via LL37 (human) or mCRAMP (mouse), which has a high binding affinity to DNA and is able to shuttle it across membranes [81]. Since it is also described as part of NETs during anti-bacterial and anti-viral defense, it is a promising molecule worth shedding further light on.

### 3.3. LL37—mCRAMP

LL37, as the only human cathelicidin, is a 37-amino-acid cationic peptide, generated by cleavage of the C-terminal end of the 18-kDa hCAP18 protein by serine proteases of the kallikrein family in keratinocytes [54,82] and proteinase 3 in neutrophils [83]. LL37 is able to form aggregates in solution and lipid bilayers and thus, unlike other antimicrobial peptides, confers protection from proteolytic degradation. Due to its positive charge, it is able to associate with negatively charged phospholipid membranes [84]. Furthermore, it has a primarily α helical shape allowing the unilateral segregation of its hydrophobic residues during membrane interactions [85]. This enables membrane penetration, formation of transmembrane pores, and bacterial lysis [85,86]. Cellular membranes associated with cholesterol, such as those found in mammals, are protected from the pore-forming effects of LL37; however, this effect can be overcome by higher concentrations of the peptide [87,88].

Exposure to LL37 results in recruitment of inflammatory cells, induction of M1 macrophages, and stimulation of inflammatory responses such as inflammasome activation and type I IFN production. Dendritic cell Type I IFN production is promoted via LL37-mediated protection of both RNA and DNA from nuclease degradation, allowing for activation of endosomal TLR7 (RNA) and TLR9 (DNA), respectively [51,52]. LL37, expressed on the surface of neutrophils, is recognized by anti-LL37 autoantibodies, which promote NET formation, generating a source of additional LL37–DNA complexes. In line with this, LL37 also contributes to protecting NET-DNA against degradation by bacterial nucleases [89]. Accordingly, LL37 has been attributed anti-microbial activities. Several murine disease models demonstrate this protective role of LL37 during bacterial as well as viral infections [90,91,92,93,94]. A recent study suggested a major role for NET-associated RNA, protected by LL37 during psoriasis, by triggering cytokine and further NET release via TLR8 and TLR13 on PMNs [53]. The newly identified component RNA within NETs, as a contributor to a self-propagating inflammatory cycle, remains to be further elucidated. 

However, studies also demonstrate anti-inflammatory effects for LL37, which are strongly dependent on the experimental setting. The antagonistic action on IFN-γ, TNF-α, IL-4, and IL-12 responses has been shown in various cell types [95,96,97]. Indeed, LL37 downregulates signaling through TLR4 via binding of its ligand, LPS [54,55], as well as through interruption of TLR4 receptor complex function in dendritic cells and macrophages [56,98]. This results in lower levels of pro-inflammatory cytokine production when LL37 and LPS are present simultaneously. Similar repression of chemokine release has been noted in epithelial cell lines [99]. In vivo, mCRAMP represses the response to 2,4-dinitrofluorobenzene-mediated contact hypersensitivity through pathways that require the TLR4 receptor [56]. Nonetheless, in a model of LPS-induced shock in mice, mCRAMP-deficiency did not significantly alter the outcome [100]. Taken together, the LL37-mediated interplay with nucleic acid and the resulting inflammatory responses warrants the further exploration of the underlying mechanisms. 

### 3.4. High-Motility Group Box 1 (HMGB1)

HMGB1 has been reported to act as a DAMP to cause sterile inflammation and is a highly conserved, non-histone chromosomal chaperone that localizes under normal physiological conditions in the nucleus of mammalian cells [101]. Human platelets, although anucleate, express HMGB1 as well [102]. Upon activation, HMGB1 localizes to the cell surface. Additionally, it can be released passively by dying cells or actively via cytoplasmic vesicles [60,103]. The passive release is rather rapid, whereas the active release is much slower [104]. During active release, HMGB1 translocates from the nucleus to the cytoplasm via JAK/STAT1-mediated acetylation. The release is at least partially mediated by double-stranded RNA-activated protein kinase R (PKR)/inflammasome-mediated pyroptosis [105]. Some studies demonstrated that HMGB1 could also be among the NETs. Following stimulation of human neutrophils with calcium phosphate-based mineralo-organic particles, NETs were released that carried HMGB1, which is relevant for the release of TNFα in co-cultured macrophages in a TLR2/4-MyD88-dependent manner [57]. In biopsies of lupus nephritis patients, it was shown that the amount of HMGB1 within NETs is elevated compared to patients without kidney disease, and it is correlated with nephritis indices such as fibrinoid necrosis, rate of glomerular filtration descent, or cellular crescents [106]. However, relatively little is known about NET-associated HMGB1, and it remains elusive whether this association is only due to its nuclear localization and DNA-binding abilities or whether it is stimulus-dependently associated with NETs with a specific task.

HMGB1-induced signaling is influenced by the redox state of three cysteines C23, C45, and C106 [107]. Fully reduced HMGB1 forms a hetero complex with CXCL12 binding to CXCR4, promoting migration of immune cells and cytokine release [58,108,109]. In contrast, fully oxidized HMGB1 does not bind to CXCR4 or TLR4 and possesses no pro-inflammatory potential [110]. Partially reduced HMGB1, also termed disulfide-HMGB1, carries a disulfide bond between C23 and C45, and can trigger inflammatory responses [111]. Once released into the extracellular space, reduced HMGB1 potentiates the inflammatory response through different mechanisms [112,113]. It is able to induce neutrophil recruitment to the site of injury [114], bind directly to PRRs such as RAGE, TLR2, TLR4, TLR9, and triggering receptor expressed in myeloid cells 1 (TREM1) [59,62,63,64,65,66], but also bind to PAMPs such as LPS [115], DNA [116] or lipoteichoic acid [117]. Following receptor ligation, a pro-inflammatory response occurs, including activation of macrophages and endothelial cells, resulting in enhanced production of pro-inflammatory chemokines, cytokines, or adhesion molecules [113]. Stimulation of platelets with thrombin, collagen, ADP, or CRP induces HMGB1-release, which in turn exerts pro-thrombotic functions [60]. Platelet HMGB1 deficiency in mice that underwent experimental trauma resulted in increased bleeding times, reduced thrombus formation, inflammation, organ damage, and platelet aggregation [60]. In vitro experiments indicate that HMGB1 is critical for regulating platelet activation, granule secretion, adhesion, and spreading in a TLR4- and MyD88-dependent manner [60,61]. Similarly, in a murine model of deep vein thrombosis, platelets accounted for the majority of HMBG1 in the circulation as well as in the development of clots. This pro-thrombotic effect was further supported and enhanced by neutrophil recruitment and NET formation, indicating that the interplay between platelet-derived HMGB1 and NET release has a crucial contribution to deep vein thrombosis in mice [118]. The induction of NET formation by platelet-derived HMGB1 was shown by Maugeri and colleagues [67]. They were able to reveal that activated platelets are able to induce NET release in a RAGE-dependent manner, whereas activated HMGB1^−/−^ platelets or the use of HMGB1 antagonists did not evoke the same effect. In a murine model of LPS-induced lung inflammation, neutrophils deriving from mice exposed to LPS and HMGB1 displayed greater ability to produce NETs compared to neutrophils isolated from mice that received LPS alone. The broncho-alveolar lavage fluids of mice treated with LPS and an HMGB1-neutralizing antibody exhibited decreased amounts of TNFα, MIP-2, histones, and cfDNA. These results indicate that HMGB1 might contribute to the production of inflammatory cytokines, as well to TLR4-mediated induction of NET release (Figure 3).

### 3.5. Cold-Inducible RNA Binding Protein (CIRP)

This recently identified DAMP is mentioned within this review, since studies suggest that it has an important role as a NET inducer and contributes to the inflammatory circle of NETs and DAMPs (Figure 3). CIRP is an 18 kDa RNA chaperone protein, originally recognized as a protein suppressing mitosis and promoting cell differentiation during hypothermia [119]. In addition to its passive release during necrotic cell death, in times of cellular stress, such as hypothermia, hypoxia, or oxidative stress, CIRP can translocate from the nucleus to cytoplasmic granules, and be further released into the environment [120,121]. Qiang and colleagues demonstrated that recombinant CIRP (eCIRP) binds TLR4 and myeloid differentiation factor 2 (MD2) and induces the release of TNFα and HMGB1 from cultured RAW264.7 cell in vitro, as well as rat macrophages in vivo [68]. Furthermore, Ode and colleagues suggested that eCIRP can induce intercellular adhesion molecule 1 (ICAM-1) expression in neutrophils, coming along with a greater ability to produce higher levels of inducible nitric oxide synthase (iNOS) and also NETs in a TLR4- and NFκB-dependent way, thereby exaggerating inflammation. Investigating *CIRP^−/−^* mice during sepsis, revealed less ICAM1^+^ neutrophils in the blood and the lungs, coming along with a significant improvement in their survival compared to wildtype (WT) mice [70]. Accordingly, NET formation and PAD4-expression were significantly decreased in the lungs of septic *CIRP*^−1^ mice compared to WT controls [69]. Further evaluating CIRP as a potent NET inducer, the same working group investigated the effect of eCIRP-induced NETs on phagocytic clearance of apoptotic cells [122]. Here, they suggest a mechanism whereby CIRP-induced NETs inhibit efferocytosis by NE-mediated cleavage of αvβ3/αvβ5 integrins in macrophages. Accordingly, during CLP-induced sepsis, *CIRP^−/−^* mice exhibited enhanced efferocytosis in the peritoneal cavity compared to WT mice. Taken together, CIRP appears to be an interesting molecule involved in NET formation and inflammation. However, further investigations from independent working groups are necessary to confirm these promising findings.

## 4. Organ Dysfunction and Remote Organ Injury

During inflammation, many components of the innate immune system act in concert to fight infection, or initiate repair mechanisms to counteract sterile injuries. As demonstrated above, there is a fragile interplay between DAMPs and the innate immune response. Studies have elaborated that NETs are possibly able to tip the scale to the inflammatory side, leading to an overwhelming immune response resulting in systemic inflammation, or cytokine storm. Those overshooting reactions might affect distant organs, which is also referred to as remote organ injury, finally resulting in multiorgan dysfunction. The following section describes impairments of organs in which NETs and NET-associated DAMPs play a pivotal role in organ dysfunction, and subsequently occurring remote organ damage. Detailed descriptions of NETs in the course of different lung or kidney diseases have recently been reviewed in more detail [123,124]. 

### 4.1. Kidney

A dysregulated immune system is often closely linked with renal diseases or injuries. Drivers of the immunopathology of renal dysfunction are versatile and comprise immune cell recruitment, formation of immune complexes, dysregulation of inflammatory mediators, immunodeficiency, autoantibody production or impaired repair mechanisms. Neutrophils and the release of NETs may contribute significantly to the pathology of renal diseases. Within this review, we focus on two renal diseases in which NETs are suggested to play an important role, not only for the underlying disease, but also for subsequently occurring complications in other organs. 

#### 4.1.1. Anti-Neutrophil Cytoplasmic Antibody (ANCA)-Associated Vasculitis

Vasculitides are inflammations in the walls of blood vessels of any organ in the body. ANCA-associated vasculitis is characterized by the lack of immune depositions (pauci-immune) and the presence of anti-neutrophil cytoplasmic antibodies (ANCAs), mostly targeting MPO and PR3, both components of NETs [125]. AAV comprise three subforms: granulomatosis with polyangiitis (GPA) [126], microscopic polyangiitis (MPA), and eosinophilic granulomatosis with polyangiitis (EGPA) [127]. GPA and EGPA mostly exhibit necrotizing granulomatous inflammation of the lower respiratory tract. GPA often affects the upper respiratory tract and can result in rhinitis, otitis, and cartilage destruction, while eosinophilia and asthma are defining features of EGPA. Renal involvement is observed in up to 90% of patients with MPA, similarly in up to 80% of patients with GPA, compared to 45% in EGPA. Accordingly, several studies have revealed NETs with NE, MPO, LL37, and PR3 in the glomeruli [128,129], or in skin lesions [130,131] and thrombi from AAV patients [132,133]. Accordingly, another study demonstrated activated tissue factor contributing to thrombosis in AAV patients [134]. During active disease, an increased incidence of venous thromboembolism in AAV patients can be observed [135,136]. Furthermore, PR3 or MPO autoantibodies are found in over 90% of patients with active disease [137], which have been suggested to harbor pathogenic functions, as further supported by results from animal models [138,139]. PR3- and MPO-ANCAs can activate neutrophils to produce ROS and proteolytic enzymes in vitro [140]. ANCA-induced neutrophil activation also leads to increased adhesion of the neutrophils [141] and the activation of the alternative complement pathway [142] with the generation of C5a. C5a in turn potentiates the inflammatory response by priming neutrophils and acting as a chemoattractant to recruit more neutrophils to the inflammatory site [143], thereby representing an inflammatory circle. Additionally, studies have revealed elevated levels of NET components, such as MPO, NE, calprotectin, PR3, and HMGB1 in the circulation of AAV patients. This might be explained by studies suggesting that neutrophils from AAV patients are more easily activated, and that PR3- and MPO-ANCA are capable of inducing NET formation in an FcR- and PR3/MPO-dependent way [144]. The high immunogenic potential of NETs was further demonstrated by injecting NET-loaded murine dendritic cells (mDCs) into recipient mice. These mice develop AAV-like disease, and exhibit circulating MPO- and PR3-ANCAs. Injection of DNAse-treated mDCs or mDCs co-cultured with apoptotic neutrophils did not evoke AAV-like disease, due to the expression of autoantibodies [131]. These observations further underline the potential of NETs and NET fragments to distribute pro-inflammatory molecules throughout the body, further exaggerating inflammation. Interestingly, in this setting, NETs link innate with adaptive immunity, indicating their relevance for autoimmune diseases. 

#### 4.1.2. Acute Kidney Injury (AKI)

AKI can be caused by many etiologies, whereas IRI belongs to the most common causes. It is characterized by a rapid decline in glomerular filtration rate and inflammation and is associated with high morbidity and mortality. Pathological presentations of AKI often include damaged tubules, dysfunctional renal vasculature, excessive inflammation, and immune cell infiltration [145]. Investigating human renal allograft biopsies with acute tubular necrosis revealed the occurrence of NETs. Ischemic AKI boosts levels of circulating and localized NETs, histones, and PAD4 expression in the affected kidneys [48]. Raup-Konsavage and colleagues demonstrated that PAD4-expressing cells are mostly neutrophils that aggregate in peritubular capillaries, interstitial space, and renal tubules after IRI [146,147]. NETs induce tubular epithelial cell death, promote clotting in peritubular capillaries via platelet–neutrophil interactions, and prime other neutrophils to form NETs [48,148]. All these events sustain hypoxia and enhance tissue damage. Interestingly, several studies demonstrate that PAD4 inhibition using pharmacological or genetic approaches protects from AKI in animal models due to a decrease in inflammation and NET formation. Meanwhile, degradation of NETs by DNase1 or anti-histone IgG also reduces renal injury, underscoring the importance of NET formation in the pathogenesis of ischemic AKI [48,147,149,150]. The pivotal role of PAD4 was further confirmed by transferring PAD4-expressing neutrophils to *Pad4^−/−^* mice, which restored NET formation in these mice and also re-sensitized them to develop AKI, indicating a pathogenic role for PAD4 or NET formation, respectively [147]. Nevertheless, PAD4 has also been connected to non-NET-related pro-thrombotic events such as the activity-reducing citrullination of a disintegrin and metalloproteinase with thrombospondin type-1 motiv-13 (ADAMTS13). It is responsible for the removal of von Willebrand factor (vWF)-platelet strings from activated endothelial cells; the relationship of vWF/ADAMTS13 is associated with an increased risk of ischemic stroke [151]. Additionally, increased levels of citrullinated antithrombin led to higher thrombin activity in patients with rheumatoid arthritis or cancer [152,153], also potentially contributing to a higher risk of vascular occlusions. Interestingly, PAD4 also citrullinates HMGB1, facilitating chromatin decondensation [154]. Whether or not citrullination of HMGB1 provides additional pro-inflammatory activity is not known yet. However, tubular necrosis and NET formation also augment remote organ dysfunction, a common feature of severe AKI, through the release of pro-inflammatory molecules, such as circulating NET-associated DAMPs such as HMGB1, and histones, or other mediators such as chemokines and cytokines [48,155]. The most frequent remote organ damage associated with AKI is Acute Lung Injury (ALI), which has a predicted mortality approaching 80% [156]. A fatal lung–kidney crosstalk occurs, and due to the extensive capillary network, the lung is highly susceptible to inflammatory mediators released by the inflamed kidney. Additionally, kidney disease is often related to a secondary immunodeficiency, which predisposes patients to secondary infections, often related to the airways and lungs [157].

### 4.2. Lung

Neutrophil recruitment to the lung is an important risk factor in the course of several infectious and non-infectious pulmonary insults. Inappropriate immune responses may turn infections into life-threatening diseases, or genetic disorders may predispose patients for chronic lung diseases. There is a strong contribution of NETs to several lung-related diseases, and the implications have recently been reviewed in detail [123]. Here, we focus on the contributions of NETs and NET-related DAMPs to respiratory dysfunction as remote organ damage, or vice versa the effect of lung injury on other organs, respectively. 

Respiratory dysfunction is characterized by hypoxemia, where the partial pressure of oxygen decreases, resulting in respiratory alkalosis and tachypnea, and thereby an increased respiratory rate in patients. Upon exposure to DAMPs, lung-resident macrophages, dendritic cells, or endothelial and epithelial cells produce inflammatory cytokines and chemokines, including TNFα, IL1, IL2, IL6, and IL8. As a consequence, the alveolar–capillary membrane permeability increases, and immune cells are recruited, further perpetuating the inflammation. Proteins leak into the interstitial tissue, resulting in alveoli injury and impaired gas exchange [158]. Several studies using different murine disease models demonstrate a pivotal role of NETs and their components on the pathogenesis of remote organ damage of the lung. Following ischemia–reperfusion of the kidney, neutrophils infiltrate the lung, increasing cytokine secretion, and NET formation, which was ameliorated upon PAD4 inhibition, thereby reducing ischemia–reperfusion-consecutive lung injury [159]. Doi and colleagues [160] revealed that ALI following AKI depends on HMGB1, which is further underlined by a recently published study demonstrating that acute lung injury following intestinal ischemia–reperfusion depends on HMGB1-induced NET formation, associated with tissue inflammation, and pathological injury in the lung [112,161]. In line with this, histones have detrimental effects on alveolar cells in vitro and in vivo during transfusion-related lung injury [75,162]. Nevertheless, a disease-dependent effect of individual histones cannot be excluded [75,163]. However, kidney–lung crosstalk may also occur, starting from lung injury, as it often occurs following mechanical ventilation. Studies using a murine model of ventilation-induced lung injury detected neutrophils and NET components in the lung microvasculature, which was proven to depend on the interaction of neutrophils and platelets [164] as well as partially on TLR4 signaling [165]. Targeting NET components by DNAse or using NE-deficient mice protected mice from lung damage, indicating that NETs contribute significantly to lung damage during ventilation-induced lung injury [164,165]. In septic patients, increased levels of complexed MPO-DNA were found, and were associated with the pathogenesis of ventilator-associated pneumonia [166]. Similarly, the damaged lung during SARS-CoV2-infection displays deformed capillaries, alveolar–capillary damage, fluid-filled alveoli, hemorrhage, fibrin deposition, signs of compensatory neovascularization, and immune cell infiltration [167,168,169,170], accounting for respiratory symptoms and shortness of breath. Disease severity was correlated with neutrophilia, indicating a direct contribution of neutrophils [171]. Sera from patients with COVID-19, exhibited elevated levels of MPO-DNA, cfDNA, and citrullinated histone H3 [167], with cfDNA and MPO-DNA being even higher in patients receiving mechanical ventilation compared to non-ventilated hospitalized patients [172]. However, the risk of AKI following mechanical ventilation is threefold higher [173], which is supposed to be induced by systemic cytokines released from injured pulmonary epithelial and endothelial cells [174,175]. A murine model of sepsis-associated ventilation indicated that ventilation may alter the expression of VEGF, VCAM-1, and angiopoietin-2 in the kidney [176]. Additionally, another study suggested that lung-derived inflammatory mediators may induce the release of inflammatory cytokines by liver endothelial cells, thereby perpetuating inflammation [177]. However, although there are several studies indicating that mechanical ventilation is associated with increased amounts of NETs, to date, there have been no studies revealing their association with remote organ damage in this setting. 

### 4.3. Liver

In terms of pathogenic conditions, there is a functional association between the liver and the lung, as well as the liver and the kidney. Dysfunction of one of these organs may cause deterioration of the other organ. It has been proposed that inflammatory mediators reaching the liver are amplified in an NFκB-dependent pathway and further released in the circulation to other organs and initiate a feed-forward mechanism of acute inflammation [177]. Additionally, the liver has a pivotal role fighting infections through the coordinated activity of neutrophils and Kupffer cells. Several studies have proven that most circulating bacteria can be efficiently cleared within the liver [178,179]. Kupffer cells can capture bacteria under flow conditions activating the complement receptor of the immunoglobulin receptor superfamily. Platelets have been suggested to fulfill a patrolling function in short-term contact with Kupffer cells, and in terms of infection with *Staphylococcus aureus* to swarm and encapsulate the caught bacteria, helping to eliminate the invaders in a vWF-dependent manner [180]. Neutrophils use expelled NETs to immobilize and eliminate pathogens from the bloodstream [29]. In contrast to other organs, liver vascular cells are able to retain NETs by anchoring them to vWF [181]. Interestingly, these anchored NETs are essentially contributing to NET-mediated injury of the respective tissue, especially through NE and histones, which are resistant to removal via DNase [181]. However, the NET-mediated early defense in the liver against invading pathogens has been proven to be indispensable for a successful immune response. Nonetheless, the long-term anchorage of the deleterious NET components harbors significant potential to perpetuate inflammation, thereby contributing to overwhelming immune responses resulting in subsequent systemic responses. This becomes evident when having a closer look on sterile liver injury. Ischemia–reperfusion injury of the liver occurs during liver surgeries, when hepatic blood supply is temporarily occluded. This results in an initial hepatocellular damage, followed by a rapid inflammatory response upon reperfusion [182,183], including the release of DAMPs, such as HMGB1, and histones [184,185,186], further inciting NET formation. In a murine liver ischemia–reperfusion model, TLR4 or TLR9 deficiency, as well as inhibition of NET formation by PAD4 inhibitors or DNase1, protected from HMGB1 and histone-mediated liver injury [49]. Accordingly, another study suggested that superoxide induces NET formation following ischemia–reperfusion in a TLR4- and NADPH-oxidase-dependent manner. Mice pretreated with allopurinol and N-acetylcysteine to decrease circulating superoxide levels exhibited decreased NET formation and improved liver injury [187]. Further studies are required to prove whether antioxidant treatment might be a valuable tool for conferring protection against NET-mediated organ injury.

### 4.4. Immunothrombosis

Thrombotic complications are among the main causes of mortality in critical ill patients. Despite the underlying injury, the factors contributing to thrombosis are versatile and very complex. Hemodynamics or blood flow in the vasculature belong to the predominant factors that dictate the rate of thrombosis by disseminating reactive components. Shear flow alterations may activate platelets and increase vWF binding and cell aggregations, leading to blockades or increased blood viscosity [188]. Additionally, the release of DAMPs or NETs can further influence hemodynamics, while also having a direct effect on thrombosis. Histones can dose-dependently enhance thrombin generation [189], and elicit thrombus formation using coagulants and triggering platelets, encouraging pro-thrombotic and pro-coagulant activity [190]. NET-associated plasma proteins such as tissue factors, fibronectin and vWF further support platelet adhesion and thrombus development [191]. Neutrophils are able to initiate thromboxane A2 production in platelets, which induces the upregulation of ICAM-1, further strengthening neutrophil–endothelium interactions [192]. Activated platelets can further fuel NET formation by vWF, platelet factor 4, and thromboxane A2 release [193], and provide the pro-inflammatory heterodimerized CXCL4 and CCL5, acting in cooperation with GPCRs and integrins [164]. Their spatial proximity is supported via anchorage of vWF to GPIb and CD11b [193]. Platelets aggregate with red blood cells into a fibrin network and attach to the damaged endothelium. Here, platelet activation and degranulation convert inactive IL-1 to the active form by thrombin cleavage, thereby connecting the coagulation system to immunothrombosis. All these mechanisms lead to the inhibition of fibrinolytic activity, thereby promoting thrombus formation and growth. Interestingly, thrombi with associated NET structures are more rigid and less permeable [194]. Many experimental animal models exist to investigate thrombosis, and the impact of extracellular DNA, not only provided by NET formation, has been reviewed in detail elsewhere [195]. 

Excessive inflammatory responses have been associated with elevated levels of interferons, interleukins, TNFs, chemokines, termed the “cytokine storm”, resulting in systemic inflammation getting along with an increased risk of thrombosis. In recent years, this deregulated immune response has gained even more attention, since it has been linked to severe manifestations of COVID-19. Here, a hypercoagulable state with thrombosis and fibrinolysis have been observed, along with high levels of D-dimer, vWF, and factor VIII [196,197]. Accordingly, platelet count, ADAMTS13, IL6, antiphospholipid antibodies, and fibrinogen were elevated [198]. Interestingly, the expression of P-selectin, as well the aggregation of platelets, neutrophils and monocytes, increased [199]. Thus, it is likely that NETs play a pivotal role during deregulated immunothrombosis and contribute significantly to respiratory diseases. Indeed, NET components such as MPO-DNA complexes are markers of disease severity in patients suffering from COVID-19 [172,200]. Microthrombi have been found in the lung, heart, and kidneys of patients with COVID-19 [200,201], and single-case autopsies exhibited NETs in the lung parenchyma and alveolar space [202,203], indicating that excessive NET formation might be a driver of COVID-19-associated intravascular coagulopathy.

Furthermore, it has been shown that NETs are associated with thrombin stimulation, fibrin clot formation, and platelet accumulation, therefore indicating the influence on the elevation of disseminated intravascular coagulation in sepsis [204]. Supporting this idea, cfDNA has been demonstrated to directly correlate with the frequency and magnitude of thrombin generation [205]. cfDNA blocks the tissue plasminogen activator, resulting in impaired fibrinolysis, and also reinforces thrombus ultrastructure by scaffolding for the binding of red blood cells, fibrin, platelets, and coagulation factors [23]. Another important factor contributing to microvascular thrombosis is HMGB1. Being also an initiator of NET formation, as component of NETs, it may be part of an inflammatory circle contributing to thrombotic events (Figure 3). In a rat model of disseminated intravascular coagulation, HMGB1 stimulated tissue factor expression in monocytes and inhibited the anticoagulant protein C pathway, mediated by the thrombin-thrombomodulin complex [103]. Interestingly, investigating a murine model of deep vein thrombosis revealed that the pro-thrombotic effect of HMGB1 was mediated through the release of extracellular DNA during NET formation [118].

## 5. NET-Targeting Therapies

Regarding the ambiguous function of NETs, it appears difficult to find the appropriate treatment. Accordingly, the published studies could not reveal a singular treatment that results in improved outcomes. However, as described above, some NET components are more noxious to the organism than others. Targeting them in preclinical studies exhibited promising results; nonetheless, currently, DNase is the only NET-targeting therapy in clinical use. Here, we summarize the most relevant and promising treatment strategies.

### 5.1. Dnase1

Extracellular chromatin and NETs can be digested by naturally occurring Dnase1. It dismantles the DNA structure and liberates entangled components, which must be calculated as a significant risk factor since, e.g., histones, NE or MPO are capable of perpetuating inflammation. To date, it has been used for the treatment of virus-associated bronchiolitis [206], as well as cystic fibrosis, in order to improve lung function and reduce the occurrence of infectious exacerbations [207,208]. Similarly, NET DNA in COVID-19 contributes to mucus accumulation, rigidity, and airway occlusion. A clinical pilot study investigated the effect of nebulized dornase-α on COVID-19 [209]. The obtained data from 10 patients treated with Dornase-α suggested a positive effect on oxygenation, which is supposed to occur through degeneration of NET complexes, as demonstrated by measuring MPO-DNA complexes. The degradation of NET structures in these patients did not result in a significant increase in secondary pulmonary infections.

### 5.2. Histones

Disentangling DNA fibers can lead to the subsequent release of histones or proteases, potentially causing cytotoxicity. As demonstrated in different murine disease models, neutralization of histones might be a promising target [210,211]. The C1 esterase inhibitor (C1INH), a serine protease inhibitor, targets multiple pathways [212,213], and due to its glycosylation-dependent overall negative charge, it is able to bind and neutralize histones. C1INH treatment reduced neutrophil activation and improved inflammation and survival in sepsis patients [214,215]. Furthermore, a recent study demonstrated that the application of anti-citrullinated protein antibody (tACPA) prevented NET-associated disease symptoms in different inflammatory pathologies in mice by inhibiting NET formation and increasing NET degradation through macrophages [211]. Another study suggested that neutralizing citH3 attenuates endothelial damage in vitro and results in improved survival rates and inflammatory responses during LPS-induced sepsis in mice [216]. Heparin is a medication, and naturally occurring glycosaminoglycan is used as an anticoagulant, able to antagonize the effects of histones [217]. Studies suggest that heparin significantly suppresses histone-induced disease [218,219], and the effect of unfractionated heparin, low-molecular-weight heparin, e.g., parnaparin, and non-anticoagulant heparin has been evaluated [219,220,221]. Here, heparin protected mice from organ damage and death by antagonizing circulating histones. Administration of heparin, especially non-anticoagulant heparin, is a novel and promising approach that requires further investigation to confirm these data. 

### 5.3. HMGB1

Studies have elaborated that anti-HMGB1 antibodies may diminish NET formation, as a reduction of H3 and cfDNA in the BALF of LPS-treated mice that received neutralizing antibodies to HMGB1 was observed [222,223]. However, blocking HMGB1 might diminish HMGB1-mediated activation of other pro-inflammatory pathways, resulting in reduced cytokine levels and therefore less immune cell recruitment. Targeting HMGB1 might be a promising approach, since it appears to play a pivotal role in the vicious circle of overwhelming inflammation during systemic diseases. Indeed, another study demonstrated that the antidiabetic drug metformin directly binds HMGB1, resulting in increased NET clearance, thereby attenuating the pro-inflammatory activity of NETs [224,225].

### 5.4. Other Treatments

There are several molecules that are able to influence NET formation. Aspirin treatment decreases NET formation in lung microcirculation and plasma [226], and also decreases the deposition of platelets with neutrophils on the lungs’ vascular walls [227]. However, a clinical study failed to verify an improved outcome following low-dose Aspirin treatment in septic patients [228]. Hydroxychloroquine, also known as an anti-malarial and anti-inflammatory drug, interferes with cytosolic sensors of nucleic acids [229,230] and inhibits the stimulation of dendritic cells by NETs via TLR9 [231]. Furthermore, it has been identified to inhibit NET formation in murine disease models [232,233]. However, its use as anti-inflammatory drug in COVID-19 patients presented controversial outcomes [234]. TLR-mediated NET formation can be inhibited by the use of blocking antibodies, such as anti-CLEC or the bispecific anti-CLEC5A/TLR2 [235]. Glucocorticoids, such as dexamethasone, belong to a class of drugs with anti-NET formation activity [236]. Furthermore, NET-inhibitory factors have been identified. They specifically inhibit NET formation in vitro and in vivo, thereby appearing to be a potential therapeutic agent [237]. Further treatment options exist that do not directly target NET formation but rather neutrophil recruitment. For example, a CXCR2 antagonist reduced neutrophil influx into the airways following an LPS challenge in humans [238]. Nonetheless, blocking neutrophil recruitment always harbors the risk of impairing the innate immune response, which might facilitate secondary infections. In this regard, a promising therapy might be the use of the CD40 antibody M7, which has been shown to limit inflammation without affecting the protective host defense in mice [239]. Nonetheless, the interplay of CD40 and its ligand CD40L was recently linked to successful resolution responses in the lung [240]. A summary of possible interventions that are targeted against NETs or their components is listed in Table 2.

## 6. Summary

The organism responds to a large variety of injuries or infections with the activation of the innate immune system. Its tasks are the switch to an inflammatory state to fight invaders, restrict or repair injuries, and finally resolve the inflammatory state and switch back to the steady state. However, although this multi-cascade process is tightly regulated, disturbances occur, and the well-balanced immune response may turn into a detrimental overwhelming inflammation. The overdosed release of cytokines or chemokines leads to the massive infiltration of neutrophils, and their subsequent activation. Neutrophil effector functions such as the release of NETs concomitantly injure surrounding tissues. Furthermore, their fragile structure, and their occurrence in the blood stream enables an easy distribution through the organism. NET-DNA, as well as NET-associated proteins, such as histones, LL37, or HMGB1, may act as DAMPs and perpetuate inflammation through the activation of PRRs and other pro-inflammatory receptors. Once distributed to other organs, they are capable of initiating further cytokine release with subsequent immune cell recruitment and activation, thereby initiating a fatal circle of pro-inflammatory mediators. Several studies have revealed a contribution of NETs to remote organ damage, hence supporting the hypothesis that NET formation may negatively influence the fine-tuned balance of the immune response towards an overshooting reaction. So far, NET-associated anti-inflammatory functions or factors contributing to the resolution of inflammation are poorly understood. However, identifying those components representing pivotal players within this circle might also provide potential targets to interrupt this pro-inflammatory circuit. To date, treatment options are scarce, and NET-directed therapies besides Dnase do not exist. Nonetheless, preclinical studies revealed that targeting NETs might be a promising strategy to reduce tissue damage, organ dysfunction, and remote organ damage, hence improving the course and outcome of many inflammatory diseases. 

## Figures and Tables

**Table 1:** DAMPs with a close association with NETs and their respective receptors.

DAMP	NET-Association	PRRs Involved	References
Histones	Part of NETs Induce NET formation	TLR2, TLR3, TLR4, TLR9	[47,48,49]
cfDNA	Part of NETs	TLR9, cGAS, IFI16, AIM2, STING	[50]
LL37	Protects NET-DNA Induction of NET formation through LL37 autoantibodies	TLR4, TLR7, TLR8, TLR9, TLR13	[51,52,53,54,55,56]
HMGB1	Induce NET formation Part of NETs	TLR2, TLR4, TLR9, CXCR4, RAGE, TREM	[57,58,59,60,61,62,63,64,65,66,67]
CIRP	Induce NET formation	TLR4	[68,69,70]

**Table 2:** Summary of NET-inhibitory compounds used for clinical and preclinical application.

Compound	Target	Model	Reference
Dornase Alfa/Dnase	DNA	Bronchiolitis Cystic fibrosis	[206,207,208]
		COVID-19	[209]
C1 esterase inhibitor	Histones	Sepsis patients	[210,214,215]
tACPA α-H3-cit	Citrullinated Histones	Inflammatory murine disease models	[211,216]
Heparin	Histones	Inflammatory murine disease models	[217,218,219,220,221]
HMGB-antibodies	HMGB1 blockade	LPS-treated mice	[222,223]
Metformin	HMGB/NET clearance	Diabetes patients	[224,225]
Aspirin (Hydroxy)chloroquine αCLEC Glucocorticoids NET-inhibiting factors	Inhibition of NET formation	COVID-19 Critically ill patients Inflammatory murine disease models	[226,227,228] [232,233,234] [235] [236] [237]
CXCR2 antagonist	Neutrophil recruitment	LPS-challenged humans	[238]
CD40L-M7	Mac1	Inflammatory murine disease models	[239]
